# A case report of hydronephrosis caused by imperforate hymen in an infant

**DOI:** 10.1097/MD.0000000000023072

**Published:** 2020-11-06

**Authors:** Meixiang Zhang, Yingchun Luo, Sheng Wang, Siyao Wang, Haiyan Kuang

**Affiliations:** a Department of Ultrasound, The Maternal and Child Health Care Hospital of Hunan Province; b Department of Ultrasound, The Children Hospital of Hunan Province, Changsha, China.

**Keywords:** hydronephrosis, imperforate hymen, ultrasound

## Abstract

**Rationale:**

Hydronephrosis, mostly caused by ureteropelvic junction obstruction, rarely occurs in infants. However, imperforate hymen atresia in female infants may cause hydronephrosis, even though it is rare.

**Patient concerns:**

A 3-month-old female infant was admitted to our hospital for frequent crying. There was no significant past medical history.

**Diagnoses:**

Following ultrasound imaging, the patient was diagnosed with hydronephrosis possibly caused by imperforate hymen.

**Interventions:**

The infant underwent hymenotomy with a cruciate incision to prevent future complications such as acute renal injury.

**Outcomes:**

Hydronephrosis resolved after the operation. The outcome was very good, with no complications in the postoperative period.

**Conclusions:**

Early ultrasound diagnosis plays a significant role in the management and treatment of infant patients. Ultrasound is the mandatory imaging technology for determining the cause of hydronephrosis.

## 1 Introduction

The common cause of hydronephrosis in infants is ureteropelvic junction obstruction, especially in the case of unilateral hydronephrosis. However, female reproductive system diseases such as the imperforate hymen could lead to pressure on the urinary system and might induce hydronephrosis. Imperforate hymen is considered as an uncommon congenital anomaly of the female reproductive tract, with an approximate incidence of 0.05% to 0.1%.^[1]^ It is detected during adolescence in most cases, presenting primary amenorrhea and lower abdominal pain.^[2]^ Although the diagnosis and treatment are simple, the disease is often delayed because of the asymptomatic nature and low incidence. There are many reports about imperforate hymen in the literature but reports about hydronephrosis in a patient with imperforate hymen are very few. A case of female infant hydronephrosis possibly caused by imperforate hymen is reported in this article. Written informed consent was obtained from the patient's parents for manuscript publication.

## 2 Case report

**Figure 1:**
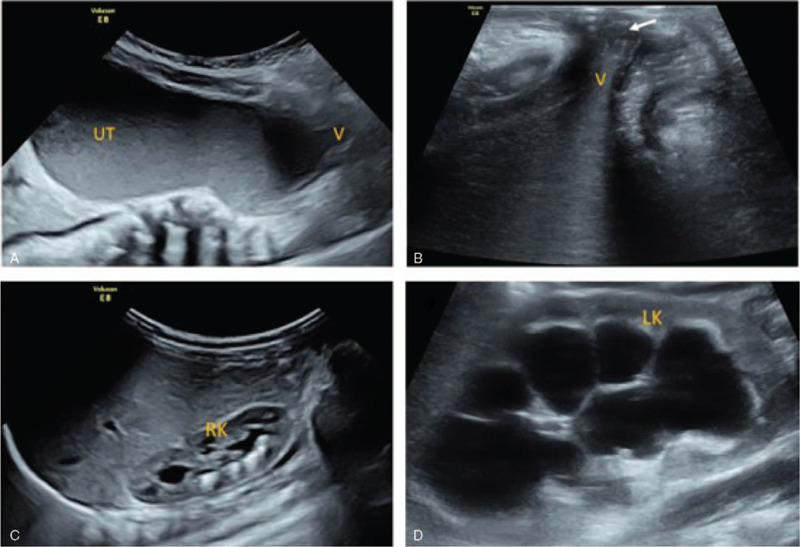
(A) Pelvic sonography showed the cystic mass was significantly enlarged (the bladder was empty). (B) Transperineal high-resolution ultrasound detected the hymen clearly (white arrow). (C) The coronal abdominal sonogram presented mild right hydronephrosis. (D) High-resolution ultrasound showed severe left hydronephrosis. LK = left kidney, RK = right kidney, UT = uterus, V = vagina.

**Figure 2:**
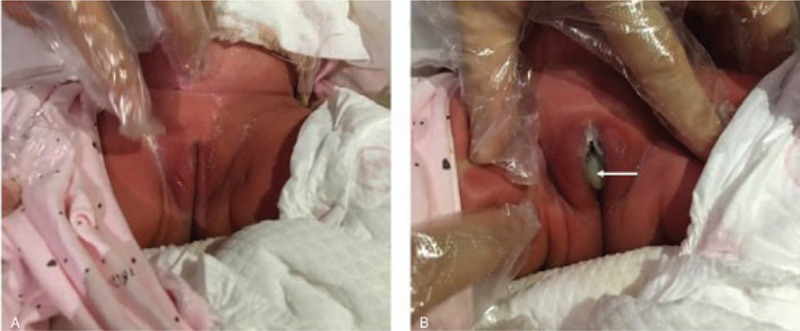
(A) The newborn had no bulging membrane in a resting state. (B) There was a bulging hymen covering the opening of vagina when she cried (white arrow).

A 3-month-old female infant was admitted to our hospital for frequent crying. Her parents were healthy, without significant family medical history. Abdominal ultrasound revealed mild right hydronephrosis and severe left hydronephrosis (Fig. 1C and D). The pelvic ultrasonography was performed after the conjecture that hydronephrosis might be caused by the lower ureter obstruction. It showed a large pear-shaped cystic mass between the bladder and the rectum with a size of 12.4 cm × 7.7 cm × 5.2 cm (Fig. 1A). The cystic mass presented as a funnel-shaped blind pouch at the distal end of the vagina with a thin membrane distended with fluid. Transperineal high-resolution sonography detected the thickness of the membrane as 0.2 cm (Fig. 1B). No vascularity was demonstrated within the cystic lesion on color Doppler flow imaging (CDFI), and the bladder was empty. In addition, the hymen was found to bulge outwards when abdominal pressure was increased during crying, which was not clearly evident in the resting state. (Fig. 2). Therefore, based on these typical ultrasound characteristics and clinical signs, the diagnosis of imperforate hymen was made. It was believed that the cystic mass might cause pressure on the ureter, resulting in hydronephrosis, especially in the left kidney. There were no abnormal laboratory values.

On account of ultrasound diagnosis, the baby underwent hymenotomy with a cruciate incision to prevent future complications such as acute renal injury. The patient tolerated the procedure well and was discharged from the hospital on the fourth postoperative day. The patient was followed-up with abdominal sonography at the local hospital after 2 months, and the follow-up results showed a mild left hydronephrosis indicating improvement.

## 3 Discussion

A sonographic diagnosis of hydronephrosis in a female infant caused by imperforate hymen is described in this article. The hymen is a thin membrane that invaginates from the perineum (urogenital sinus) to meet the longitudinal vaginal canal (a Müllerian structure).^[3]^ The hymenal orifice could establish a channel between the vagina and the exterior for egress of cervical gland secretions and eventually, menstrual blood products. It could bring about imperforate hymen when the hymen fail to perforate during the later stages of embryonic development.^[4,5]^ Generally speaking, most patients with imperforate hymen are diagnosed with amenorrhea during adolescence after menarche. The prenatal sonogram findings of imperforate hymen usually appear as a pear-shaped pelvic cystic mass between the bladder and the rectum.^[6]^

Once the pelvic cystic mass gradually increases, it causes mechanical pressure on the urinary tract, resulting in bladder outlet obstruction and hydronephrosis.^[7]^ However, in the reported literature, prenatal ultrasonography examination showed patients with imperforate hymen having normal kidneys, with hydronephrosis occurring after birth.^[8,9]^ Although mild hydronephrosis is often physiological, severe hydronephrosis may lead to renal obstruction.^[10]^ Other potential etiologies of hydronephrosis include posterior urethral valves, ureteral obstruction, and urolithiasis. In addition, most of the mild hydronephrosis subsides naturally, while severe hydronephrosis may develop worsening renal function such as renal failure. Moreover, hydronephrosis is often considered a risk factor for the loss of renal function.^[11]^ Therefore, it is critical to identify the cause of hydronephrosis and start treatment. Because of the accessibility, reasonable price, and non-invasiveness with non-ionization radiation, ultrasound is considered as the preferred imaging method for diagnosing and monitoring hydronephrosis. Ultrasound played a vital role in diagnosis and management in our case too.

Although imperforate hymen is an unusual cause of hydronephrosis, it is still necessary to perform renal sonography for every patient with imperforate hymen to assess whether there are complications, so that timely treatment can be given. Meanwhile, careful inspection of the infant's external genitalia is essential. The diagnosis of the imperforate hymen early in life is critical for counseling and timely intervention. Therefore, future severe complications such as acute kidney injury could be prevented. Presently, the diagnosis of hydronephrosis mainly relies on ultrasonography, a mandatory imaging technology in diagnosing the cause of hydronephrosis and monitoring it.

## Author contributions

**Conceptualization:** Meixiang Zhang, Haiyan Kuang.

**Investigation:** Sheng Wang, Siyao Wang.

**Funding acquisition:** Yingchun Luo, Haiyan Kuang.

**Supervision:** Haiyan Kuang.

**Writing – original draft:** Meixiang Zhang.

**Writing – review & editing:** Yingchun Luo, Haiyan Kuang.
